# Functional Characterization of Potato *UBC13*-*UEV1s* Genes Required for Ubiquitin Lys63 Chain to Polyubiquitination

**DOI:** 10.3390/ijms24032412

**Published:** 2023-01-26

**Authors:** Weigang Liu, Xun Tang, Xue Fu, Huanhuan Zhang, Cunlan Zhu, Ning Zhang, Huaijun Si

**Affiliations:** 1 State Key Laboratory of Aridland Crop Science, Gansu Agricultural University, Lanzhou 730070, China; 2 College of Agronomy, Gansu Agricultural University, Lanzhou 730070, China; 3 College of Life Science and Technology, Gansu Agricultural University, Lanzhou 730070, China

**Keywords:** potato, UEVs, UBC13, gene expression, protein interaction

## Abstract

Ubiquitin-conjugating enzymes (E2s/UBC) are components of the ubiquitin proteasome system (UPS), and the ubiquitin-conjugating enzyme variant (UEV) is one of E2s (ubiquitin-conjugating enzymes, UBC) subfamily. The UEVs and UBC13 play an auxiliary role in mediating Lys63-linked polyUb chain assembly, which is correlated with target protein non-proteolytic functions, such as DNA repair or response to stress. However, the collaborative mechanism of *StUBC13* (homologue of AtUBC13) and *StUEVs* (the UEVs in potato) involved in potato are not fully understood understood. Here, we identified two *StUBC13* and seven *StUEVs* from potato genome. We analyzed protein motif and conserved domain, gene structure, phylogenetic features, *cis*-acting elements of *StUBC13* and *StUEVs*. Subsequently, we screened *StUBC13* partners protein and verified interaction between *StUBC13* and *StUEVs* using yeast two-hybrid, split luciferase complementation (SLC) and bimolecular fluorescence complementation (BiFC) approach. The expression profile and qRT-PCR analysis suggested that *StUBC13* and *StUEVs* gene exhibited a tissue-specific expression and were induced by different stress. Overall, this investigative study provides a comprehensive reference and view for further functional research on *StUBC13* and *StUEV1s* in potato.

## 1. Introduction

The ubiquitin-proteasome system (UPS), a post-translational modification (PTM), is responsible for selectively removing most of the regulatory proteins of eukaryotes cells (80–90%) [1]. The UPS plays a critical role in virtually every aspect of the cytological and physiological processes of plant cells. Ubiquitin (Ub) is well established as a reusable recognition signal for protein degradation or reversible, non-proteolytic regulatory events. Ubiquitination proceeds through a sequential three-step enzymatic cascade reaction from activation to transfer (ubiquitin-activating enzyme E1→ubiquitin-conjugating enzymes E2→ubiquitin ligase enzymes E3), which results in Ub or Ub polymers covalently bonding to target proteins.

The -COOH group from the ubiquitin (Ub) C-terminus, activated by an ATP-dependent reaction, conjugates with the E1 Cys residue to form a stable intermediate E1~Ub (ATP~Ub→E1~Ub) via a high-energy thioester bond. Then, E1~Ub transfers Ub to the Cys residue of the E2 active site through a transesterification reaction to form the E2~Ub complex (E1~Ub→E2~Ub). E3 recruits the ubiquitin-loaded E2 enzyme into proximity with the target protein and facilitates the formation of isopeptide bonds between the ε-amino group of the target protein lysine (K) and the glycine from the Ub C-terminus. As a result, E3 ligase is responsible for recognizing the substrate, followed by either direct ubiquitin transfer (RING or U-box, CRLs) or thioester formation (HECT) with ubiquitin. In this process, the target protein is ubiquitinated by one or more Ub units (monoubiquitination or multi-monoubiquitination) or a Ub chain (defined as polyubiquitination).

Ubiquitin possesses several modification sites, Lys6, Lys11, Lys27, Lys29, Lys33, Lys48 Lys63, and Met1 (N-terminal methionine) to form distinct homo- and heterotypic polyubiquitin chains [2]. Modified with Lys11, Lys29, and Lys48 polyUb chains are potent signals for target proteins degradation by the 26S proteasome [2,3]. Monoubiquitylation and Lys63-linked Ub polymers are implicated in nonproteolytic outcomes in plants, such as hormonal responses and development [4]. However, evidence from other studies suggests that Lys63 polyUb polyubiquitination may serve as a signal for target protein degradation by the 26S proteasome [5]. Lys63-linked polyUb chains are the second most abundant type of ubiquitin linkage with proteasome-independent functions in plants, and are involved in plant development, hormonal responses, nutritional responses, biotic interactions, DNA repair, and responses to environmental conditions [6].

E2 possesses a core UBC domain that contains a Cys active site mediating the ubiquitination of the target protein. UBC13, the only E2 enzyme identified that facilitate Lys63 polyUb chain, functions require a collaborator protein, a UBC E2 variation known as MMS2 or UEV1 [7]. Typically, UBC13 and UEV1/MMS2 form a heterodimer to catalyze the synthesis of K63 polyUb chains. In this process, the UBC13 active site Cys provides the binding site for the first Ub, whereas UEV1/MMS2 E2 non-covalently binds the second Ub to polymerize the Lys63-linked chain extension [8]. UBC13 and UEV1s have been identified and functionally characterized in several plants, indicating that they are conserved in terms of sequence and function [9]. Four Arabidopsis UEV1s (AtUEV1A-D) and rice UEV1s (OsUEV1A-D) were identified [10,11], which interacted with UBC13 (UBC13A, UBC13B). UBC13 diverges to undertake multiple functions in Arabidopsis, such as apical dominance, iron metabolism to promote development response, auxin signaling to affect root development, and response to low temperature and pathogens [9]. UEV1s fluctuate in different tissues and during abiotic stress at transcription level [12]. Recent studies have revealed that OsUEV1B is required to maintain Pi homeostasis in rice [13]. UBC13 interacts with UEV1s to form a stable heterodimer to assembly of Lys63-linked polyubiquitin chains, which play a critical role in error-free DNA-damage tolerance and repair [14]. In addition, UBC13 and UEV1s complexes coordinately assemble and transfer Lys63-linked polyubiquitin chains to target proteins through E3. AtUBC13A/AtUEV1B interacts with AtRGLG1 and AtRGLG2, which carries Lys63-linked Ub chains to AtPIN2 for regulating polar auxin transport and adaptive growth responses [6]. However, UBC13 and UEVs have not been characterized, and little is known about the roles of UBC13-UEV1s complexes in potato.

Here, we reported that seven *StUEVs* and two *StUBC13* from potato, of which *StUBC13* and *StUEV1s* were further described in molecular cloning and functionally characterized. We also exclusively screened *StUBC13* partners and verified their interactions relationship. Moreover, RNA-seq data and qRT-PCR were used to analyzed expression patterns of *StUBC13* and *StUEVs*. This study contributes to further explore functional diversity and molecular mechanisms of *StUBC13* and *StUEVs* in potato.

## 2. Results

### 2.1. Identification and Characterization of Potato UEVs

The integrity of the UBC domain (lack of Cys active site, UEV domain) was confirmed by InterProScan and SMART programs. There are seven *StUEVs* in potato which are named based on being matched to the genes of Arabidopsis UEVs: *StUEV1B*, *StUEV1D-like*, *StUEV1D-like1*, *StCOP10*, *StVPS23/ELC*, *StELC-like*, and *StELC-like2*. The potato *UEVs* were disproportionately distributed on chromosomes 1, 4, 9, and 10. The longest ORFs were *StELC-like*, encoding 407 amino acid residues. The StUEVs genes information is listed in Table 1.

**Figure 1:**
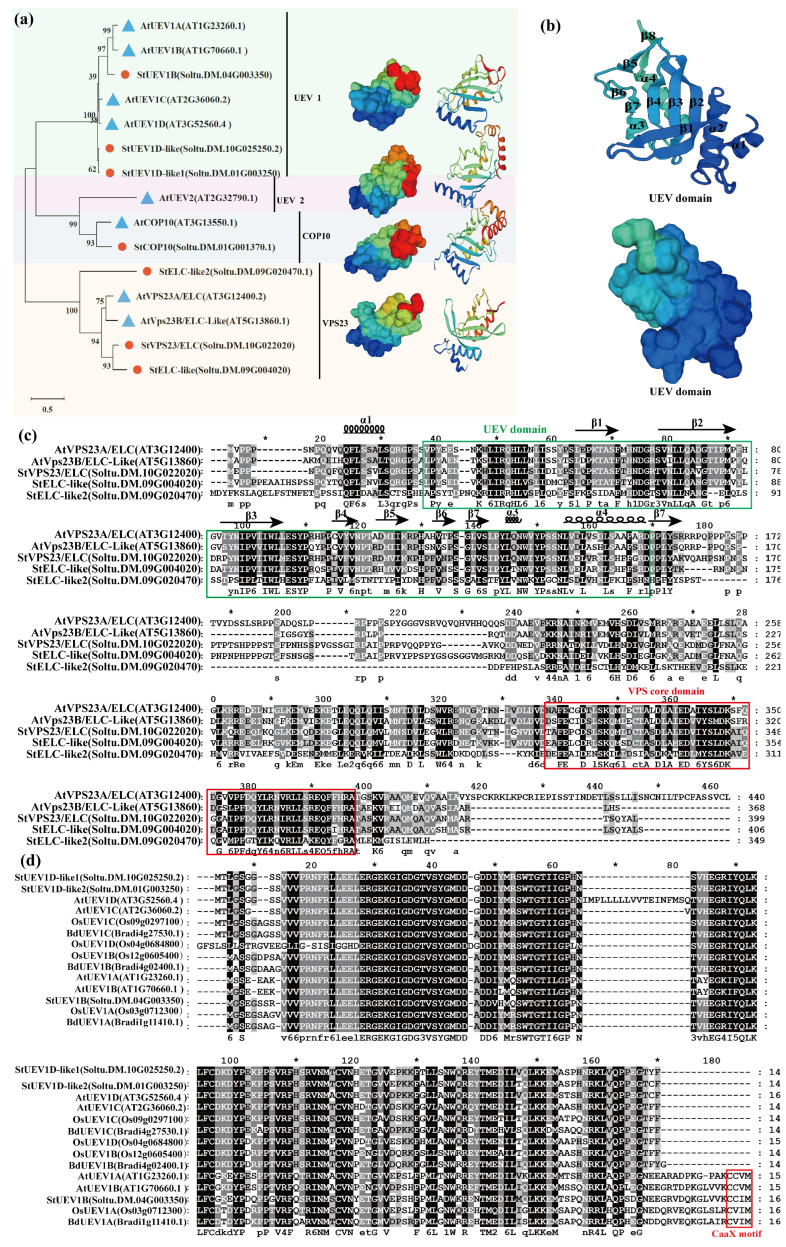
Characterization analysis of UEVs gene family. (**a**) Phylogenetic relationships and classification of *UEVs* gene family. (**b**) The three-dimensional structure of UEVs conserved domain. (**c**) Protein sequence alignment of VPS23 subgroups in Arabidopsis and potato. (**d**) Protein sequence alignment of UEV1s subgroups from *Oryza sativa*, *Arabidopsis thaliana*, *Brachypodium distachyon*, and *Solanum tuberosum*.

Classification and phylogenetic relationships showed that *StUEVs* are classified into four groups (Figure 1a), potato *StUEVs* are distributed in three subgroups, as well as lack of StUEV2: StUEV1s (StUEV1B, StUEV1D-like, StUEV1B-like1), StCOP10 (StCOP10), StVPS23 (StVPS23/ELC, StELC-like, StELC-like2). In addition, StUEV1A and StUEV1C are absent in the StUEV1 subfamily, while StUEV1D is expanded. In the StVPS23 subgroups, StELC-like2 forms a distinct evolutionary branch and expands the StVPS23 subfamily. The analysis of spatial structure of its homologue AtUEVs showed that different *StUEVs* are identical in the spatial configuration of UEV conserved domain but varied in other areas apart from UEV conserved domain in different subgroups (Figure 1b). To further investigate the functions of StUEVs, multiple sequence alignments were conducted on the conserved UBC domain (Figure 1c). The StVPS23 subfamily consists of N-terminal UEV and C-terminal VPS domain, among which the UEVs domain is comprised of α helices, 8 β sheets, and a few random coils. The StUEV1s subfamily contains a conserved UEV domain, while the C-terminus is variable. Most UEV1s have a TXY conserved motif at the C-terminus. However, the AtUEV1A/AtUEV1B/StUEV1B/OsUEV1A/BdUEV1A have feature C-terminal extensions with the CaaX motif (Figure 1d). UEV domains are present in both the Arabidopsis and potato sequences of the StCOP10 subfamily, with a similarity of 61.54%.

### 2.2. The Cis-Regulatory Elements Analysis

**Figure 2:**
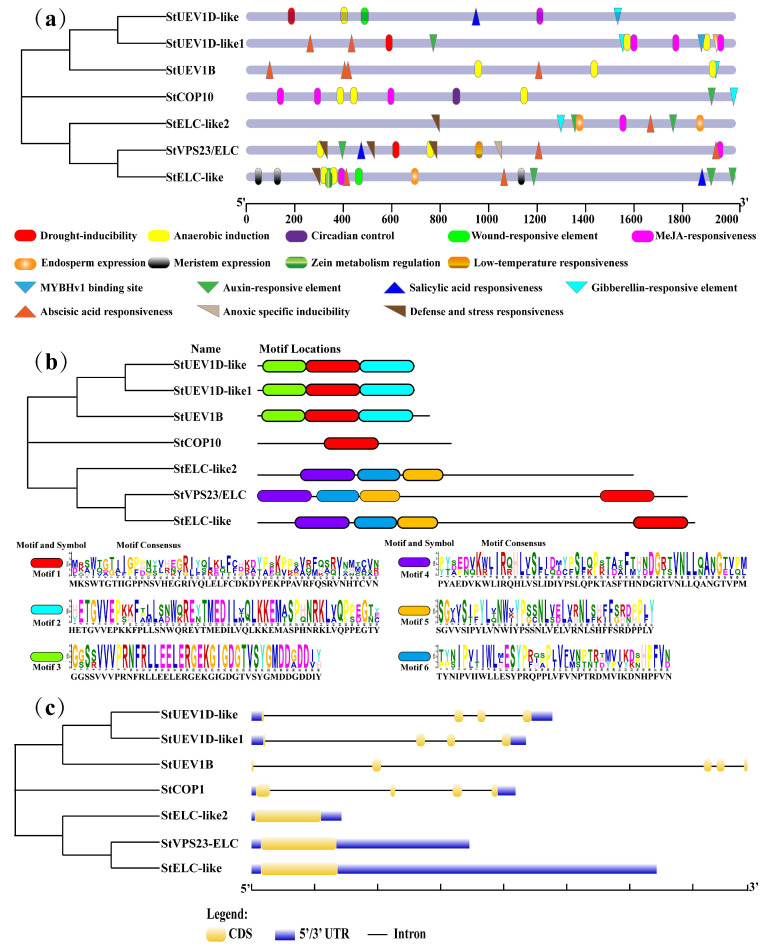
The *cis*-acting element, conserved motif, and gene structure analysis of UEVs. (**a**) The *cis*-acting element analysis of *StUEVs* genes in the promoter region. (**b**) The motif composition analysis of *StUEVs*. (**c**) The gene structure analysis of *StUEVs*.

Analysis of the *cis*-acting elements showed that a variety of the elements related to hormone pathways, such as, such as abscisic acid ABA (ABRE), gibberellin GA (P-box and GARE-motif), salicylic acid SA (TCA-element), auxin IAA (TGA-box/element), and methyl jasmonate MeJA (TGACG-motif and CGTCA-motif), of which six *StUEVs* contain *cis*-acting elements associated with MeJA responses (Figure 2a, Table S1). Five *StUEVs* contain ABA/GA/IAA responses associated with *cis*-acting elements, suggesting that most *StUEVs* are related to MeJA, ABA, GA, and IAA signaling pathways.

### 2.3. Conserved Motif and Gene Structure Analyses

The motifs in the same subfamily are similar in type and distribution (Figure 2b). The types and distributions of motifs shown in different subgroups vary greatly, particularly between the VPS23 and UEV1s subgroups. The UEV1 subgroups have three distinct motifs, motif1, motif2, and motif3. The VPS23 subgroups contains three different specific motifs, including motif4, motif5, and motif6. In addition, motif1 was widely distributed in *StUEVs*, while only StELC-like2 is absent of motif1. Interestingly, StCOP1 contains only motif1. To better understand gene structural diversity, we examined the exon–intron distribution patterns of *StUEVs* genes. The *StUEVs* occupied positions in the same clade with similar intron-exon distribution patterns. UEV1 and COP1 subgroups were spliced with three introns and four exons, while StUEV1B contains four introns and five exons. Particularly, the VPS23 subgroups have no introns. These results suggest that *StUEVs* motifs and gene structure display diverse distribution patterns. (Figure 2c).

### 2.4. Identification and Characterization of Potato StUBC13

Based on a BLAST search, two *StUBC13s* were identified in potato, which were named corresponding to the Arabidopsis genes: *StUBC35*/*StUBC13A*, *StUBC35*/*StUBC13A-like* (Table S2). *StUBC13A* and *StUBC13A-like* are located on chromosome 10 and 7, respectively. There are segmental duplication events of the *StUBC13* gene in potato, indicating that the *StUBC13* gene participates in the evolution of large gene families.

**Figure 3:**
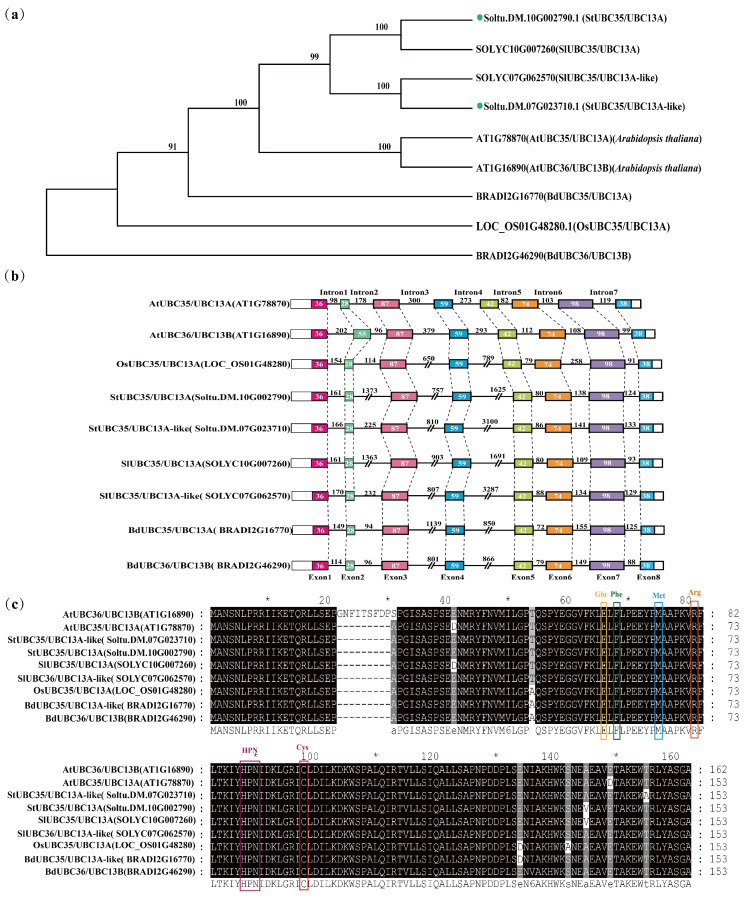
The characterization of *StUBC13*. (**a**) Phylogenetic relationships of UBC13 from *Oryza sativa*, *Arabidopsis thaliana*, *Brachypodium distachyon*, *Solanum lycopersicum*, and *Solanum tuberosum*. (**b**) Gene structure analysis of UBC13. (**c**) Protein sequence alignment of UBC13. * indicates that this residue is conserved.

Phylogenetic tree analysis revealed that the *StUBC13s* of five plants descended from the same branch and were orthologs (Figure 3a). Some species lost *UBC13B* throughout the evolution of *UBC13s* (such as tomato, potato, and rice), whereas others retained *UBC13B*. (such as *Brachypodium*). *StUBC13A* evolved from a common plant ancestor and is the closest relative to *SlUBC13s* of tomato and the most distant relative to *BdUBC13B* of *Brachypodium*.

Gene structure analysis showed that *StUBC13s* were consistent with the number and distribution of intron-exon (Figure 3b). However, the length of the second exon (53 bp) of *AtUBC13B* was different from that of other species (28 bp). The lengths of other corresponding exons were consistent, and the nucleic acid sequences were highly conserved. The intron length and sequence identity at the same location varied greatly between species, while the intron lengths of *StUBC13A* and *SlUBC13A*, *StUBC13A-like*, and *SlUBC13A-like* at the same site were nearly identical. The sequence identity was the highest, 88.55%, indicating that *UBC13s* were highly conserved not only in the exon sequence but also in the intron sequence during potato and tomato evolution. The promoter region analysis showed that several *cis*-acting elements participate in in abiotic stress and hormones responsiveness (Table S1).

Compared with the amino acid sequence of the UBC13s of other organisms (Figure 3c), the active site Cys87 formed with the Ub thioester, the Met64 interacting with RING-type E3 ligase, and Glu55, Phe57 and Arg70 interacting with Mms2 (UEV1s) are conserved [9]. In comparison to other plants, the protein sequence of Arabidopsis thaliana AtUBC13B contains nine amino acid residues (GNFITSFDP) inserted between positions 21–29, and the S amino acid at position 30 differs from the A amino acid in other species.

### 2.5. StUBC13 Interacted with StUEV1s and E3 Ligase Enzyme StRGLG1

**Figure 4:**
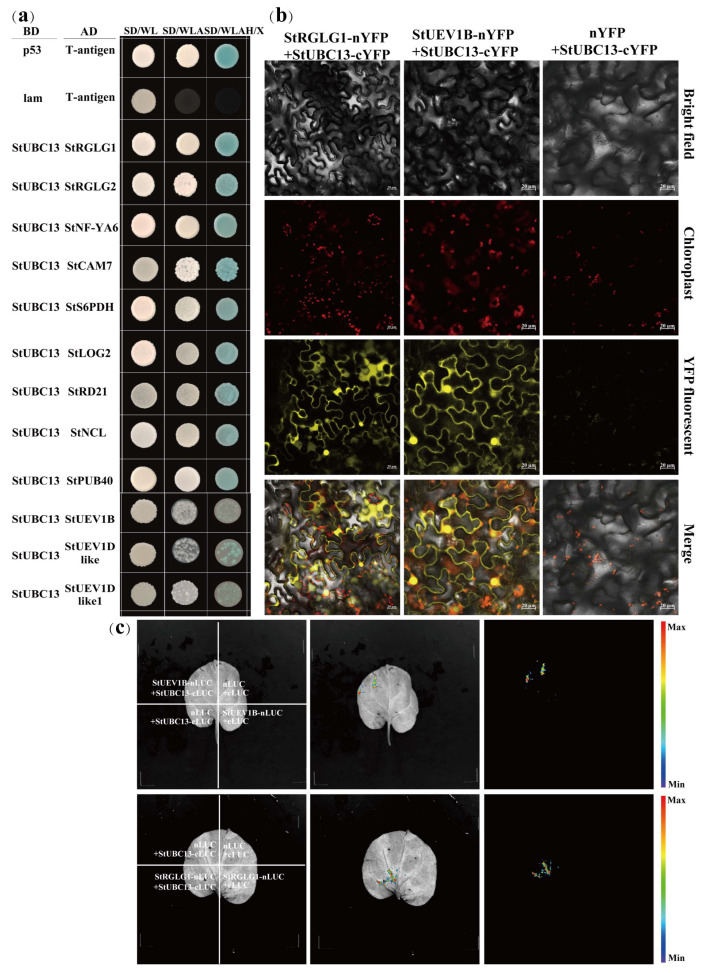
*StUBC13* interacts with multiple proteins. (**a**) The verification of *StUBC13* and multiple protein interactions by one-to-one Y2H. (**b**) The interaction between StRGLG1, StUEV1B and *StUBC13* in BiFC assay. (**c**) The interaction between StRGLG1, StUEV1B and *StUBC13* in SLC assay.

To further clarify the working mechanism of *StUBC13* in potato, we screened StUBC13A partners by the yeast two-hybrid method. Twenty-five positive clones were identified, including StUEV1B, and a RING-type E3 ligase enzyme StRGLG1 (Table S3). Among these candidates, we selected nine partners and then performed one-to-one yeast two-hybrid (Y2H) assays to check the physical interactions between *StUBC13* and its partners. Our results showed that the StRGLG1/2, StNF-YA6, StCAM7, StS6PDH, StLOG2, StRD21, StNCL, and StPUB40 candidates interacted with *StUBC13* in the yeast two-hybrid assay (Figure 4a). We were mainly interested in SUEV1s/StRGLG1/2/StNF-YA6/StCAM7/StS6PDH/StLOG2/StRD21/StNCL/StPUB40, which work with *StUBC13* to play a critical role in the biological process. To confirm whether *StUBC13* interacts with StUEV1B/StRGLG1/2, StNF-YA6/StCAM7/StS6PDH/StLOG2/StRD21/StNCL/StPUB40, the full CDS length of *StUBC13* was constructed into the pGBKT7 vector as bait, the full CDS length of StRGLG1/2/StNF-YA6/StCAM7/StS6PDH/StLOG2/StRD21/StNCL/StPUB40 were inserted into pGBKT7 vector as prey, respectively. We verified that *StUBC13* interacted with StRGLG1/2/StNF-YA6/StCAM7/StS6PDH/StLOG2/StRD21/StNCL/StPUB40 in the yeast cell by one-to-one Y2H assay.

BiFC and SLC assays were conducted to further verify StUBC13-StUEV1B and StUBC13-StRGLG1 interactions. The BiFC assay (bimolecular fluorescence complementation) showed that YFP fluorescence signals were observed in the nucleus and cell membrane in epidermal cells, which indicated that *StUBC13* and StUEV1B interactions occurred in the nucleus, cytoplasm, and cell membrane (Figure 4b). We observed a specific reconstituted luciferase activity signal using SLC (split-luciferase complementation) assays when the combination of *StUBC13* and StUEV1B/StRGLG1 was coexpressed in *N. benthamiana* leaves (Figure 4c). These data confirm that *StUBC13* interacted with StUEV1B and StRGLG1 in vivo, respectively.

The GO analysis revealed that *StUBC13* partners were significantly enriched to the biological process of response to stimulus (GO:0050896) (Figure S1a). There was a significant statistical overlap between five biological processes of *StUBC13* and its targets, including protein metabolic process (GO:0019538), cellular protein modification process (GO:0006464), response to chemicals (GO:0042221), and response to stress (GO:0006950). In addition, KEGG pathway annotation showed that UBC13 partners were significantly enriched in metabolism, genetic information processing, environmental information processing, cellular processing, and organismal system (Figure S1b).

### 2.6. Subcellular Localization of UBC13 and Its Partners

**Figure 5:**
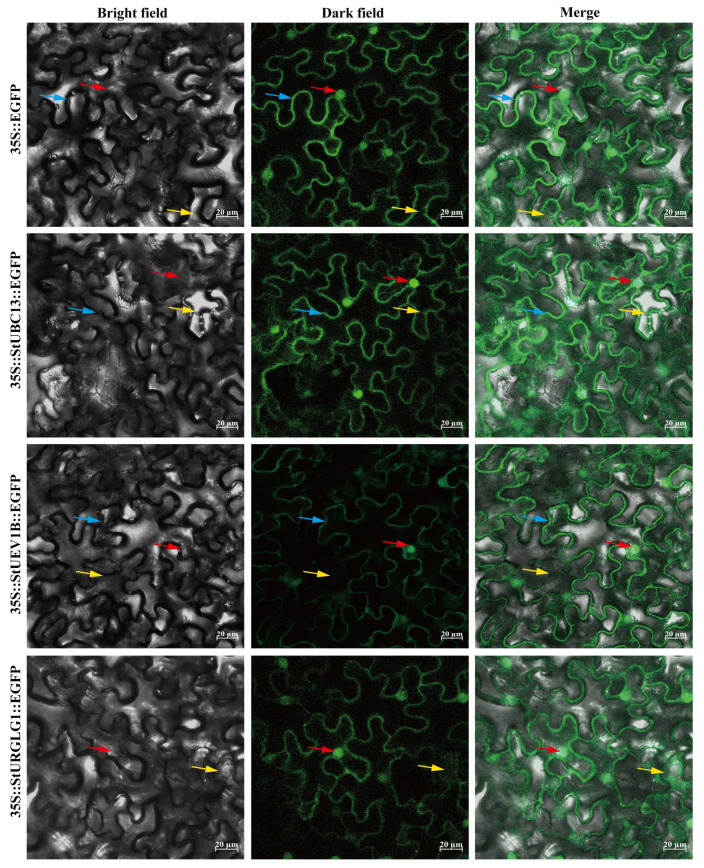
Subcellular localization of *StUBC13* and its partners. The red arrows indicate the nucleus, the blue arrows the cell membrane, and the yellow arrows indicate membrane.

To further explore the role of *StUBC13* in plant cells, *StUBC13* CDS was fused with green fluorescence protein (eGFP) to produce fluorescence signals for subcellular localization. The sample was expressed in tobacco (*N. benthamiana*) leaves using the Agrobacterium-mediated infiltration method. Confocal imaging showed that 35S: *StUBC13*-EGFP was distributed in the nucleus and cell membrane, and continuous intensity fluorescent on the cell membrane (Figure 5). Similarly, StUEV1B and RGLG1 also observed GFP fluorescence signals in the nucleus, cytoplasm, and cell membrane (Figure 5). These results indicate that *StUBC13*, StUEV1B, and StRGLG1 were localized in the nucleus, cytoplasm, and cell membrane.

### 2.7. Expression Patterns of StUEVs and StUBC13

**Figure 6:**
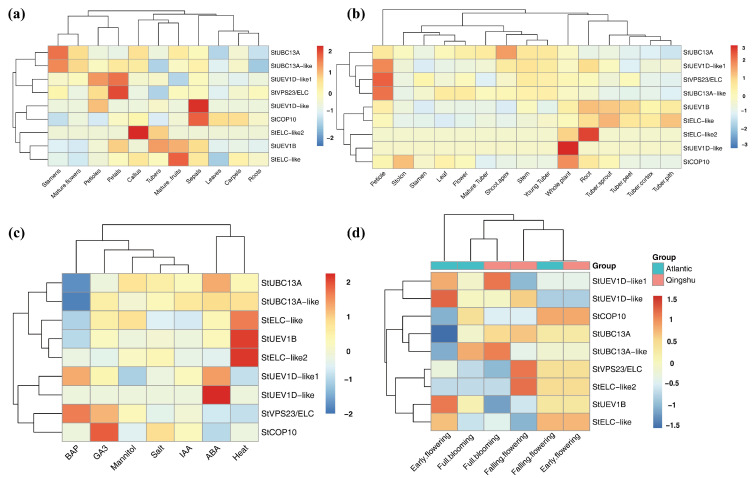
The expression patterns of *StUEVs* and *StUBC13*. (**a**) The tissue-specific heatmap of *StUEVs* and *StUBC13* in DM. (**b**) The tissue-specific heatmap of *StUEVs* and *StUBC13* in RH. (**c**) The expression patterns of *StUEVs* and *StUBC13* under different treatments in DM. (**d**) The expression patterns of *StUEVs* and *StUBC13* in “Atlantic” and “Qingshu” during the flowering stage under drought stress.

To further reveal the role of *StUEVs* and *StUBC13*, RNA-seq data was performed to analyze the tissue-specific expression level. There are different tissue-specific expression patterns in DM and RH. *StUBC13A* and *StUBC13A-like* in stamens, *StUEV1D-like1* and *StVPS23/ELC* in petals, *StELC-like2* in callus, *StELC-like* in mature fruits, and *StCOP10* and *StUEV1D-like1* in sepals displayed significant expression levels in DM, respectively (Figure 6a). *StUEV1D-like1*, *StVPS23/ELC*, *StUBC13A-like* in the petiole, StUBC13A in the shoot apex, *StELC-like2* in root had the highest expression level in RH (Figure 6b). Other genes were not differentially expressed among tissues in DM and RH. The different expression profiles showed the various roles of *StUEVs* and *StUBC13* in potato development and growth.

To further understand the function of *StUEVs* and *StUBC13*, the publicly available transcriptome data were performed an expression profile analysis of *StUEVs* and *StUBC13* gene after drought, heat, mannitol, salt, ABA, IAA, GA3, BAP (Figure 6c). *StELC-like2*, *StUEV1B*, and *StELC-like* were obviously upregulated after heat stress. The expression levels of *StUEV1D-like*, *StUEV1D-like1*, and *StUBC13A* were higher under ABA stress. The transcript levels of *StCOP10* and *StVPS23/ELC* were significantly higher under GA3 stress. *StUEV1D-like1* and *StVPS23/ELC* were slightly upregulated following BAP stress. In addition, *StUBC13A* and *StUBC13A-like* were noticeably downregulated after BAP stress. All had no obvious expression changes under salt, ABA, mannitol stress. For drought stress, the expression levels of *StUEV1D-like*, *StUEV1D-like1*, *StUEV1B*, *StELC-like* were significantly induced in the early flowering Atlantic, while *StUBC13A*, *StUBC13A-like*, *StCOP10* were less expressed in the early flowering Atlantic (Figure 6d). *StVPS23/ELC* and *StELC-like* were highly expressed in the falling flowering Qingshu, *StUEV1D-like1* and *StUBC13A-like* were obviously expressed in the full flowering Qingshu.

### 2.8. The qRT-PCR Analysis of StUEVs and StUBC13 in Potato

**Figure 7:**
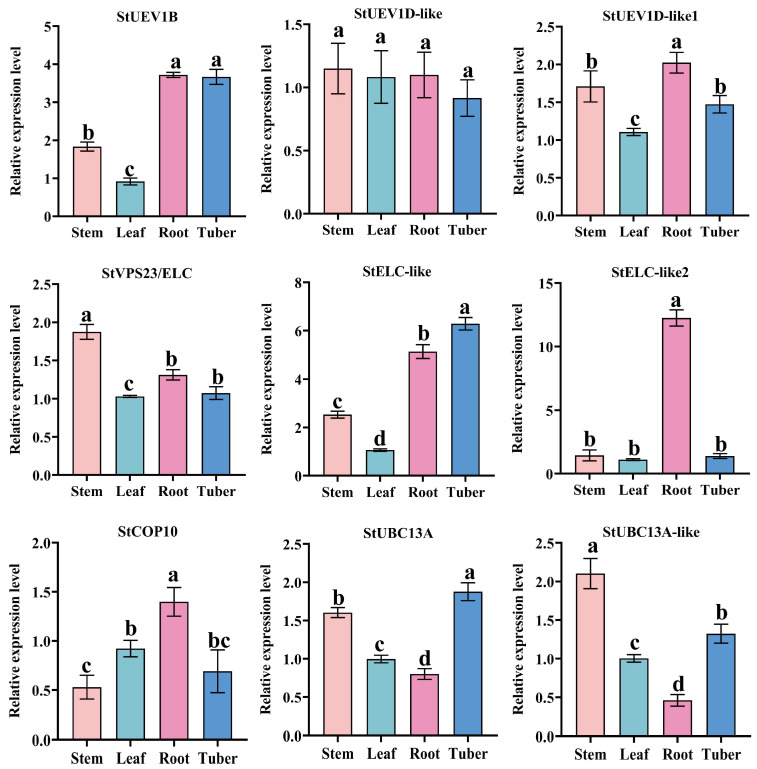
The qRT-PCR analysis of *StUEVs* and *StUBC13* in different potato tissues. Different small letters indicate statistical differences of tissues when assessed using Duncan’s multiple range test (*p* < 0.05, *n* = 3).

To further determine the expression patterns of *StUEVs* and *StUBC13s*, qRT-PCR was performed to analyze organ-specific expression levels in Atlantic (Figure 7). *StUEV1D-like1*, *StUEV1B*, *StELC-like*, and *StELC-like2* in root had the highest transcript abundances. *StUEV1B*, *StELC-like*, and *StUBC13A* in tubers exhibited significant expression levels. *StUEV1D-like1*, *StVPS23/ELC*, *StUBC13A*, *StUBC13A-like* in stems had a significant expression level. Interestingly, *StUEV1D-like* was not specifically expressed between tubers, stems, roots and leaves. These results suggested that *StUEVs* and *StUBC13* play an important role in potato development and growth.

**Figure 8:**
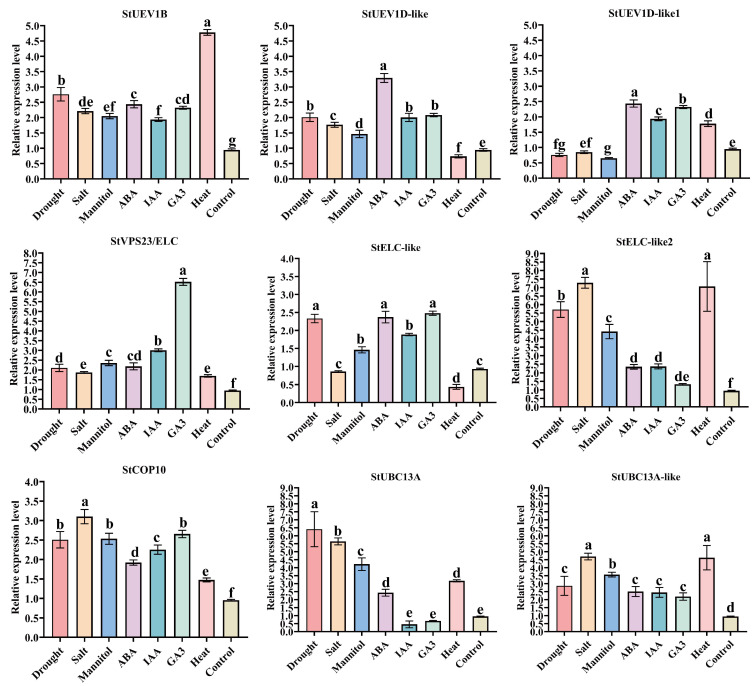
The qRT-PCR analysis of *StUEVs* and *StUBC13* under different stress. Different small letters indicate the statistical differences of tissues when assessed using Duncan’s multiple range test (*p* < 0.05, *n* = 3).

To further explore the role of *StUEVs* and *StUBC13* in potato, the expression patterns of *StUEV1s* and *StUBC13* were determined by qRT-PCR under different stresses (Figure 8). All genes showed different expression patterns under various stresses. *StUEV1B*, *StCOP10*, *StVPS23/ELC*, *StUBC13A-like* had asignificantly upregulated expression under all stress treatment. *StUBC13A* and *StELC-like* had enhanced expression after drought stress. The expression level of *StELC-like2*, *StCOP1*, *StUBC13A*, StUBC13A-like were significantly increased under salt stress. All genes were up-regulated under ABA treatment. *StUEV1D-like*, and *StELC-like* were down-regulated under heat stress. *StUEV1D-like1* was down-regulated under drought, salt, and mannitol stress, but was up-regulated by ABA, IAA, GA3, and heat stress. This suggests *StUEV1s* and *StUBC13* were implicated in different stresses.

Overall, *StUEVs* and *StUBC13* participated in potato development and growth, and responses to different types of stress.

## 3. Discussion

Lys63-linked polyubiquitination is widely considered to be a non-canonical ubiquitation and is different from conventional Lys-48-linked polyubiquitination. The Lys63-linked polyubiquitination process is a signal for a nonproteolytic rather than mediating the target protein degradation by 26S proteasome. Lys63-linked polyubiquitination is similar to phosphorylation and sumoylation, which influence target protein activities to play a critical role in several cellular processes. UBC13, the only known E2, can catalyze Lys-63-linked Ub chain assembly by forming stable heterodimers complex with UEV1s [15,16]. In this study, *StUBC13A* in potato was isolated and characterized in potato. The gene structure analysis showed that the distribution pattern of UBC13s introns/exons was highly conserved in different species (Figure 3b) [17,18]. The phylogenetic analysis and sequence alignment of amino acid was 98.69% identified between *StUBC13A* and *StUBC13A-like* (Figure 3a), which were separated from one gene duplication events [19]. Both tomato and potato have two *StUBC13A*, indicating that these two species have expanded StUBC13A during evolution, which is consistent with previous research on UBC13 gene duplication events (PGDD: http://chibba.agtec.uga.edu/duplication/index/locus, accessed on 22 September 2022). Therefore, *StUBC13* genes were involved in genome duplication or gene fusion in potato during the evolution of the E2 gene family, which indicates that *StUBC13* genes may be functionally redundant, consistent with previous studies [20]. Of great interest is the observation that the expression of *StUBC13s* is induced by different stress (Figure 8), which is similar to the *BdUBC13A* results in *Brachypodium* [20]. However, *UBC13* was considered a housekeeping gene in both Arabidopsis and rice [17].

To date, *UEVs* have been identified in several plants, including eight *UEVs* in *A. thaliana*. However, only four and three *UEV1s* subfamilies have been identified in rice (*O. sativa*) and *B. distachyon*, respectively [11,21]. In this study, we characterized seven highly conserved *StUEVs* in potato (Table S1). The phylogenetic analysis showed that members of the *StUEVs* family were expanded/lost in potato during the evolution process, such as the loss of the UEV2 and the expansion of the VPS23 subgroups (Figure 1a). This suggests that, during the evolutionary process of adapting to the environment, *StUEVs* might respond to environmental changes and needs by selecting, eliminating, or extending their members. Sequence alignment analysis showed that StUEV1B included a special C-terminal extension containing the CaaX motif except for the typical UEV conserved domain (Figure 1c), which is similar to AtUEV1A, AtUEV1B, OsUEV1A, BdUEV1A sequence feature. The CaaX motif is considered to be the isoprene-specific site in plants, which plays a crucial role in the functional differentiation between AtUEV1A/B and AtUEV1C/D and affects the subcellular localization to confer other nuclear functions [12,21]. Similarly, we propose that the CaaX motif may be implicated in functional differentiation between StUEV1B and StUEV1D-like/StUEV1D-like1 and subcellular localization (Figure 5). The distribution pattern and number of introns/exons and motifs is in accordance with in the same subfamily (Figure 2b,c), which implies little diversity between the same subgroups [10]. *StUEV1s* exhibited distinct expression patterns in different potato tissues (Figure 6 and Figure 7). AtUEV1s are ubiquitously expressed in Arabidopsis, and *AtUEV1D* is highly expressed in shoots, which is consistent with the expression profiles of *StUEV1B*. Previous studies showed that *AtUEV1s* were derived from a segmental duplication gene, and the duplication events result in similarity expression profiles and conserved functions [10]. A *cis*-acting element in the *StUEVs* gene promoter region serves as a binding site for transcription factors and regulates gene expression. Distinct *cis*-acting components serve different purposes. qRT-PCR analysis showed that the *StUEVs* family specifically expressed in different tissue and induced expression under different stresses (Figure 8). This is associated with the *cis*-acting elements of hormonal and abiotic stress responses in the *StUEVs* promoter region (Figure 2a). Therefore, *UEVs* are involved in response to abiotic stress and development [10,13].

The formation of Lys63-linked polyubiquitination is required to the stabilize the heterodimer complex UBC13-UEV1s, which is involved in the regulation of DNA damage and response to stress. UBC13 interacted with Uev1s to form stable heterodimers, which confers different functions [21]. In this study, *StUBC13* interacted with all three StUEV1s (Figure 4a), which suggested that StUEV1s and *StUBC13* may be involved in DNA damage and response to stress [11]. Several partners of *StUBC13* were screened by the yeast two-hybrid, including StRGLG1/2, StNF-YA6, StCAM7, StS6PDH, StLOG2, StRD21, StNCL, and StPUB40 (Figure 4a), of which homologous partners play a crucial role in regulating plant growth and development [22,23,24,25,26,27,28,29]. The GO and KEGG analysis showed that the partners were involved in metabolism and response to stimulus (Figure S1). For example, RGLG1/2 ubiquitin E3 ligase can interact with *StUBC13* to form a E2-E3 complex in potato using Y2H, SLC, and BiFC technology assays, and the complex is involved in the iron deficiency response of Arabidopsis roots [30]. In this regard, future research will focus on how StUBC13-StUEV1s conjugates different E3s to form Lys-63-linked poly-Ub chains and influences target proteins activities to regulate multiple cellular processes, such as [8,31,32,33,34,35].

## 4. Materials and Methods

### 4.1. Identification and Phylogenetic Analysis of StUBC13s and StUEVs

The Arabidopsis and rice UBC13 and UEVs sequences were obtained from the Arabidopsis information source (TAIR) database (http://www.arabidopsis.org, accessed on 9 September 2022) and Rice Genome Annotation Project Database (https://rice.plantbiology.msu.edu/, accessed on 9 September 2022), which were used to search *StUBC13* and *StUEVs* using BLAST search in SpudDB (http://spuddb.uga.edu/,accessed on 12 September 2022) [36]. The candidate proteins were further verified by InterProScan (http://www.ebi.ac.uk/Tools/pfa/iprscan, accessed on 9 September 2022) [37] and SMART (http://smart.embl-heidelberg.de/, accessed on 9 September 2022) [38] to confirm the existence of the UBC/UEV conserved domain. The UBC13 and UEVs protein sequences were aligned using Clustal software (http://www.clustal.org/, accessed on 15 September 2022) and then constructed a phylogenetic evolutionary tree using MEGA X with the neighbor-joining method (NJ) according to 1000 bootstrap replicates, respectively. Subsequently, UEVs were divided into four subgroups according to the Arabidopsis UEVs classification [15].

### 4.2. Multiple Sequence Alignment, Gene Structure and Motif, and Cis-Acting Elements Analysis

The protein sequences of UBC13 and UEVs from several species were performed to multiple sequence alignment to analyse conserved domain features by ClustalW and visualized using GENEDOC [39]. The distribution of the exon–intron structure UEVs in potato were examined by Gene Structure Display Server (GSDS: http://gsds.cbi.pku.edu.cn, accessed on 15 September 2022). The UEVs conserved motifs analysis was performed by MEME online program (https://meme-suite.org/meme/doc/meme.html, accessed on 15 September 2022) [40]. The UEVs subfamily of the promoter regions (from 0 to −1000 bp of coding sequence) was performed to analyze cis-acting elements using the PlantCARE database (http://bioinformatics.psb.ugent.be/webtools/plantcare/html/, accessed on 17 September 2022).

### 4.3. Yeast Two-Hybrid Assay

The yeast two-hybrid (Y2H) assay was performed as previously described [41]. The *StUBC13* gene (*StUBC13A*) was recombined into the pGBKT7 vector as bait and used to screen the potential interaction proteins of *StUBC13* according to instructions from Clontech. The StUEV1s gene were cloned into the pGADT7 vector as prey and were used to verify the interaction between *StUBC13* and StUEV1s through co-transforming into yeast strain YH109 by one-to-one Y2H assay. The primer used in this study is listed in Table S4.

### 4.4. Gene Ontology (GO) and KEGG Analysis

To further correlate UBC13 with its partners, the Gene Ontology (GO) terms and KEGG were identified and enriched using OmicShare tools, a free online data analysis (https://www.omicshare.com/tools, accessed on 22 September 2022).

### 4.5. Subcellular Localization and BiFC, SLC Analysis

The *StUBC13*, StUEV1B and StRGLG1 gene were fused to the pCAMBIA1300-EGFP vector, respectively. Similarily, *StUBC13*, StUEV1B and StRGLG1 were separately recombined into the pSPYCE and pSPYNE vectors, and the pCAMBIA1300-CLuc and pCAMBIA1300-NLuc vectors. The different combinations were transformed into *Nicotiana benthamiana* leaves, respectively. The GFP and YFP fluorescence signal were observed by confocal microscope (Zeiss LSM780, Oberkochen, Germany). The LUC recombinant fluorescence signal was imaged by PlantView 100 (Guangzhou Biolight Biotechnology, Guangzhou, China). The primer used in this study is listed in Table S1.

### 4.6. Expression Profiles of StUBC13s and StUEV1s

The raw transcriptome data was downloaded from the publicly available transcriptome at NCBI (Project ID: PRJNA541096) and SpudDB (DM and RH potato) [36]. The RNA-seq data was analyzed and visualized using TBtools [42].

### 4.7. Plant Materials and Growth Condition

The potato cultivar Atlantic was cultured in MS medium supplemented with 0.75% agar and 3% sucrose under 22 °C, 16 h normal light/8 h dark. After 4 weeks of growth, the plants were transplanted into pots with soil mix (10 × 10 cm), and then placed in a growth chamber at 21 ± 2 °C under 16 h light/8 h dark and watered weekly. After another 4 weeks, the plants were classed into nine groups to be subjected to different treatments and controls groups. For the salt and mannitol stress treatment, the plants were irrigated with 200 mM NaCl and mannitol for 24 h, while the controls were irrigated with water. For hormone treatment, the plants leaves were sprayed with 50 µM ABA, 10 µM IAA, and 50 µM GA3 for 24 h, respectively, while the controls were sprayed with water. For the drought stress treatment, the plants were cultured without water to reach 25 ± 5% of soil field capacity, while the controls were watered weekly. For the heat stress treatment, the plants were stored at 35 °C for 24 h, while the controls were grown in normal condition. The differently treated samples were harvested and immediately frozen in liquid nitrogen, and stored at −80 °C.

### 4.8. RNA Extraction and qRT-PCR Assay

The total RNA of the samples was extracted by using the RNAprep Pure Plant Kit (Tiangen Biotechnology, Beijing, China) and then synthesized to first strand of cDNA by FastKing RT Kit (Tiangen Biotechnology, Beijing, China). The gene expression level was detected by qRT-PCR using SuperReal PreMix Plus (SYBR Green) (Tiangen Biotechnology, Beijing, China) with LightCycler^®^ 96 System. The reference gene *Stef1α* (GenBank accession: AB061263) was used for data normalization. The results were calculated using the 2^−△△CT^ method [43].

## 5. Conclusions

UBC13 and UEVs were required for the ubiquitin Lys63 chain to undergo polyubiquitination. In this study, we systematically identified and analyzed *StUBC13* and *StUEVs* gene structure, conserved domains, cis-acting elements, interactions, and gene expression in potato. Our observations collectively suggest that *StUBC13* interacted with StUEV1s to form complexes that may be involved in multiple cellular processes.

## Figures and Tables

**Table 1:** List of the identified *StUEVs* genes and related information.

Gene Name	Accession Number	Protein/AA^1^	Chrom^2^	MW (Da)^3^	pI^4^	Instability Index	GRAVY^5^
StUEV1B	Soltu.DM.04G003350	161 AA	chr4:3,714,877–3,722,749	19,476.32	9.25	34.47	−0.617
StUEV1D-like	Soltu.DM.01G003250	147AA	chr1:3,436,712–3,441,067	16,560.81	6.20	29.72	−0.601
StUEV1D-like1	Soltu.DM.10G025250	147AA	chr10:56,826,223–56,830,996	16,648.90	6.20	30.24	−0.601
StVPS23/ ELC	Soltu.DM.10G022020	400AA	chr10:54,196,275–54,199,731	44,902.08	6.07	47.44	−0.434
StELC-like	Soltu.DM.09G004020	407AA	chr9:3,420,331–3,426,759	45,550.78	6.17	48.10	−0.451
StELC-like2	Soltu.DM.09G020470	350AA	chr9:55,278,138–55,279,565	40,160.35	4.97	50.05	−0.336
StCOP10	Soltu.DM.01G001370	181AA	chr1:1,515,501–1,519,690	19,476.32	9.25	34.36	−0.222

AA^1^, amino acid residues; Chrom^2^, chromosome; MW^3^, molecular weight; pI^4^, isoelectric point; GRAVY^5^, grand average of hydropathicity.

## Data Availability

Not applicable.

## Supplementary Materials

The following supporting information can be downloaded at: https://www.mdpi.com/article/10.3390/ijms24032412/s1.
