# Supplementary material for: Functional Characterization of Potato UBC13-UEV1s Genes Required for Ubiquitin Lys63 Chain to Polyubiquitination
Source: Int J Mol Sci. 2023 Jan 26;24(3):2412. doi: 10.3390/ijms24032412 (PMC9917286; doi:10.3390/ijms24032412)
Supplement: Supplementary file 1 [file ijms-24-02412-s001.zip › ijms-2149287-supplementary.pdf]

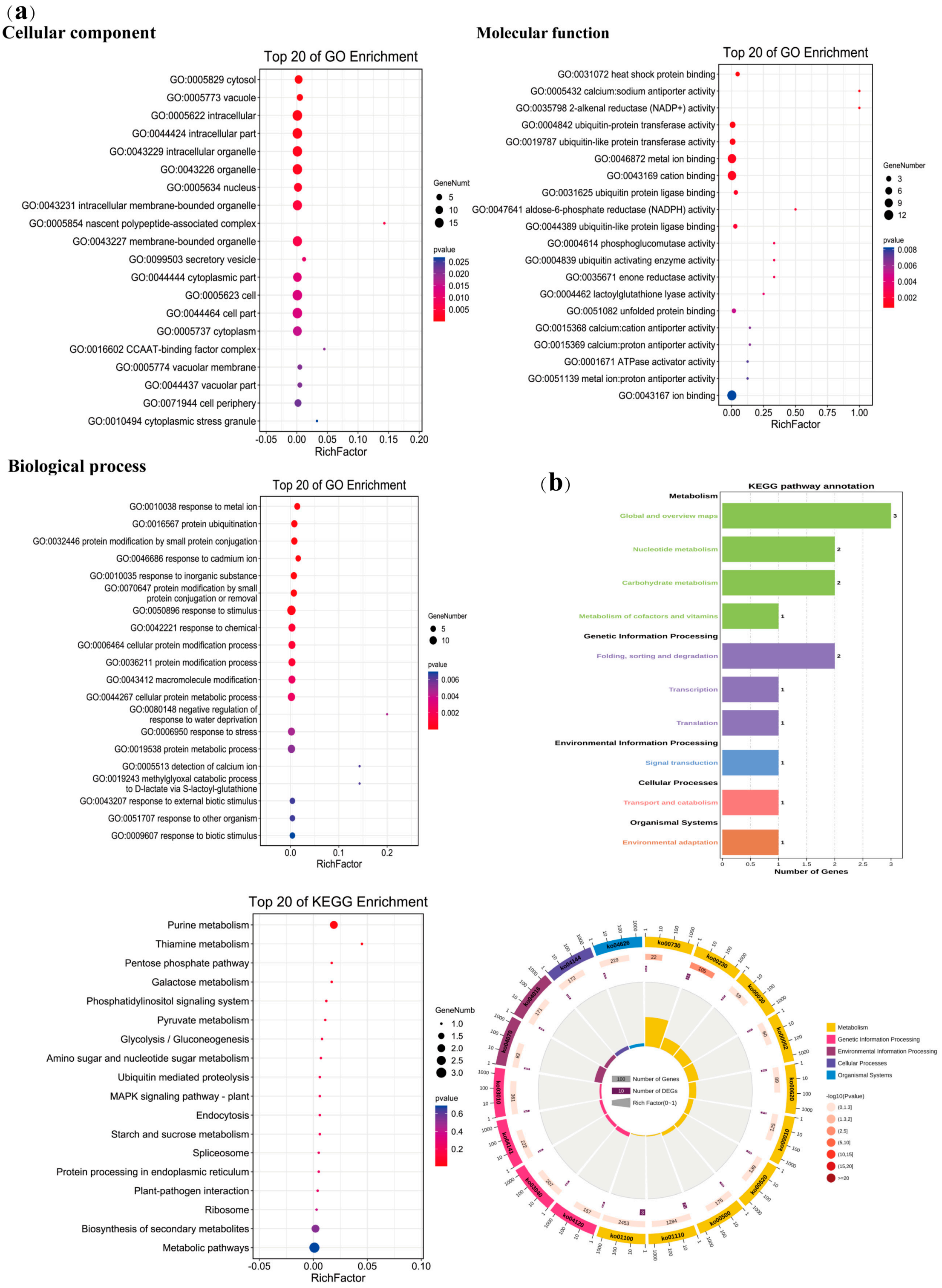

Figure S1 The GO and KEGG analysis of StUBC13 interactors. (a) The GO analysis. (b) The KEGG analysis.

Table S1 List of the *cis*-acting element information.

Gene	motif	sequence	position	Strand	Function
StCOP10	CGTCA-motif	CGTCA	133	-	cis-acting regulatory element involved in the MeJA-responsiveness
	CGTCA-motif	CGTCA	287	+	cis-acting regulatory element involved in the MeJA-responsiveness
	CGTCA-motif	CGTCA	592	-	cis-acting regulatory element involved in the MeJA-responsiveness
	circadian	CAAAGATATC	862	-	cis-acting regulatory element involved in circadian control
	ARE	AAACCA	396	-	cis-acting regulatory element essential for the anaerobic induction
	ARE	AAACCA	452	+	cis-acting regulatory element essential for the anaerobic induction
	ARE	AAACCA	1160	+	cis-acting regulatory element essential for the anaerobic induction
	TGACG-motif	TGACG	133	+	cis-acting regulatory element involved in the MeJA-responsiveness
	TGACG-motif	TGACG	287	-	cis-acting regulatory element involved in the MeJA-responsiveness
	TGACG-motif	TGACG	592	+	cis-acting regulatory element involved in the MeJA-responsiveness
	P-box	CCTTTTG	2014	-	gibberellin-responsive element
	TGA-element	AACGAC	1923	+	auxin-responsive element
StELC-like	ABRE	ACGTG	389	+	cis-acting element involved in the abscisic acid responsiveness
	ABRE	ACGTG	1061	+	cis-acting element involved in the abscisic acid responsiveness
	WUN-motif	AAATTTCCT	457	-	wound-responsive element
	TCA-element	CCATCTTTTT	1882	-	cis-acting element involved in salicylic acid responsiveness
	AuxRE	TGTCTCAATAAG	1919	-	part of an auxin-responsive element
	O2-site	GATGA(C/T)(A/G)TG(A/G)	385	+	cis-acting regulatory element involved in zein metabolism regulation
	TGACG-motif	TGACG	387	+	cis-acting regulatory element involved in the MeJA-responsiveness
	CGTCA-motif	CGTCA	387	-	cis-acting regulatory element involved in the MeJA-responsiveness
	GCN4_motif	TGAGTCA	689	+	cis-regulatory element involved in endosperm expression
	LTR	CCGAAA	314	-	cis-acting element involved in low-temperature responsiveness
	TGA-element	AACGAC	1184	-	auxin-responsive element

	TGA-element	AACGAC	2080	-	auxin-responsive element
	TC-rich repeats	GTTTTCTTAC	295	+	cis-acting element involved in defense and stress responsiveness
	ARE	AAACCA	325	+	cis-acting regulatory element essential for the anaerobic induction
	ARE	AAACCA	369	-	cis-acting regulatory element essential for the anaerobic induction
	CAT-box	GCCACT	41	-	cis-acting regulatory element related to meristem expression
	CAT-box	GCCACT	120	+	cis-acting regulatory element related to meristem expression
	CAT-box	GCCACT	1134	+	cis-acting regulatory element related to meristem expression
StELC-like2	GCN4_motif	TGAGTCA	1360	-	cis-regulatory element involved in endosperm expression
	GCN4_motif	TGAGTCA	1877	+	cis-regulatory element involved in endosperm expression
	TATC-box	TATCCCA	1299	+	cis-acting element involved in gibberellin-responsiveness
	TGA-element	AACGAC	1355	-	auxin-responsive element
	TGA-element	AACGAC	1763	+	auxin-responsive element
	TC-rich repeats	GTTTTCTTAC	791	+	cis-acting element involved in defense and stress responsiveness
	ABRE	ACGTG	1670	+	cis-acting element involved in the abscisic acid responsiveness
	TGACG-motif	TGACG	1557	+	cis-acting regulatory element involved in the MeJA-responsiveness
StUEV1B	ABRE	CACGTG	86	+	cis-acting element involved in the abscisic acid responsiveness
	ABRE	ACGTG	87	+	cis-acting element involved in the abscisic acid responsiveness
	ABRE	ACGTG	398	+	cis-acting element involved in the abscisic acid responsiveness
	ABRE	TACGGTC	411	-	cis-acting element involved in the abscisic acid responsiveness
	ABRE	ACGTG	1205	-	cis-acting element involved in the abscisic acid responsiveness
	P-box	CCTTTTG	1939	+	gibberellin-responsive element
	ARE	AAACCA	969	+	cis-acting regulatory element essential for the anaerobic induction
	ARE	AAACCA	1451	-	cis-acting regulatory element essential for the anaerobic induction
	ARE	AAACCA	1944	-	cis-acting regulatory element essential for the anaerobic induction
	MBS	CAACTG	178	+	MYB binding site involved in drought-inducibility

StUEV1D-like	ARE	AAACCA	412	-	cis-acting regulatory element essential for the anaerobic induction
	P-box	CCTTTTG	1532	+	gibberellin-responsive element
	GA-motif	ATAGATAA	474	+	part of a light responsive element
	WUN-motif	AAATTTCCT	481	-	wound-responsive element
	TCA-element	CCATCTTTTT	942	+	cis-acting element involved in salicylic acid responsiveness
	TGACG-motif	TGACG	1211	+	cis-acting regulatory element involved in the MeJA-responsiveness
	CGTCA-motif	CGTCA	1211	-	cis-acting regulatory element involved in the MeJA-responsiveness
StUEV1D-like1	GARE-motif	TCTGTTG	1553	-	gibberellin-responsive element
	ARE	AAACCA	1575	+	cis-acting regulatory element essential for the anaerobic induction
	ARE	AAACCA	1919	+	cis-acting regulatory element essential for the anaerobic induction
	CCAAT-box	CAACGG	1897	+	MYBHv1 binding site
	MBS	CAACTG	584	-	MYB binding site involved in drought-inducibility
	TGA-element	AACGAC	766	-	auxin-responsive element
	CGTCA-motif	CGTCA	1602	+	cis-acting regulatory element involved in the MeJA-responsiveness
	CGTCA-motif	CGTCA	1776	-	cis-acting regulatory element involved in the MeJA-responsiveness
	CGTCA-motif	CGTCA	1946	+	cis-acting regulatory element involved in the MeJA-responsiveness
	TGA-box	TGACGTAA	1943	-	part of an auxin-responsive element
	TGACG-motif	TGACG	1602	-	cis-acting regulatory element involved in the MeJA-responsiveness
	TGACG-motif	TGACG	1776	+	cis-acting regulatory element involved in the MeJA-responsiveness
	TGACG-motif	TGACG	1946	-	cis-acting regulatory element involved in the MeJA-responsiveness
	ABRE	ACGTG	255	+	cis-acting element involved in the abscisic acid responsiveness
	ABRE	ACGTG	427	+	cis-acting element involved in the abscisic acid responsiveness
	GC-motif	CCCCCG	1035	-	enhancer-like element involved in anoxic specific inducibility
	ARE	AAACCA	313	-	cis-acting regulatory element essential for the anaerobic induction
	ARE	AAACCA	770	+	cis-acting regulatory element essential for the anaerobic induction

StVPS23/ELC	TC-rich repeats	GTTTTCTTAC	315	+	cis-acting element involved in defense and stress responsiveness
	TC-rich repeats	GTTTTCTTAC	521	-	cis-acting element involved in defense and stress responsiveness
	TC-rich repeats	ATTCTCTAAC	764	+	cis-acting element involved in defense and stress responsiveness
	LTR	CCGAAA	958	+	cis-acting element involved in low-temperature responsiveness
	TGA-element	AACGAC	388	+	auxin-responsive element
	MBS	CAACTG	612	-	MYB binding site involved in drought-inducibility
	CGTCA-motif	CGTCA	1959	-	cis-acting regulatory element involved in the MeJA-responsiveness
	TGACG-motif	TGACG	1959	+	cis-acting regulatory element involved in the MeJA-responsiveness
	TCA-element	TCAGAAGAGG	465	+	cis-acting element involved in salicylic acid responsiveness
	ABRE	CACGTG	1204	-	cis-acting element involved in the abscisic acid responsiveness
	ABRE	ACGTG	1205	+	cis-acting element involved in the abscisic acid responsiveness
	ABRE	ACGTG	1941	+	cis-acting element involved in the abscisic acid responsiveness
StUBC13A	ABRE	AACCCGG	1057	+	cis-acting element involved in the abscisic acid responsiveness
	ARE	AAACCA	1081	-	cis-acting regulatory element essential for the anaerobic induction
	ARE	AAACCA	1086	-	cis-acting regulatory element essential for the anaerobic induction
	ARE	AAACCA	1096	-	cis-acting regulatory element essential for the anaerobic induction
	ARE	AAACCA	1101	-	cis-acting regulatory element essential for the anaerobic induction
	ARE	AAACCA	1136	-	cis-acting regulatory element essential for the anaerobic induction
	ARE	AAACCA	1141	-	cis-acting regulatory element essential for the anaerobic induction
	ARE	AAACCA	1157	+	cis-acting regulatory element essential for the anaerobic induction
	ARE	AAACCA	1173	+	cis-acting regulatory element essential for the anaerobic induction
	ARE	AAACCA	1467	-	cis-acting regulatory element essential for the anaerobic induction
	ARE	AAACCA	1854	-	cis-acting regulatory element essential for the anaerobic induction
	ARE	AAACCA	1859	-	cis-acting regulatory element essential for the anaerobic induction
	ARE	AAACCA	1894	-	cis-acting regulatory element essential for the anaerobic induction

	ARE	AAACCA	1899	-	cis-acting regulatory element essential for the anaerobic induction
	LTR	CCGAAA	1791	+	cis-acting element involved in low-temperature responsiveness
	LTR	CCGAAA	1801	+	cis-acting element involved in low-temperature responsiveness
	LTR	CCGAAA	1809	+	cis-acting element involved in low-temperature responsiveness
	LTR	CCGAAA	1818	+	cis-acting element involved in low-temperature responsiveness
StUBC13A-like	A-box	CCGTCC	968	+	cis-acting regulatory element
	ABRE	ACGTG	648	+	cis-acting element involved in the abscisic acid responsiveness
	ABRE	ACGTG	1278	+	cis-acting element involved in the abscisic acid responsiveness
	ABRE	ACGTG	1541	-	cis-acting element involved in the abscisic acid responsiveness
	ARE	AAACCA	1079	+	cis-acting regulatory element essential for the anaerobic induction
	ARE	AAACCA	1561	-	cis-acting regulatory element essential for the anaerobic induction
	ARE	AAACCA	1918	-	cis-acting regulatory element essential for the anaerobic induction
	RY-element	CATGCATG	1912	-	cis-acting regulatory element involved in seed-specific regulation
	P-box	CCTTTTG	2147	-	gibberellin-responsive element
	HD-Zip 1	CAAT(A/T)ATTG	933	+	element involved in differentiation of the palisade mesophyll cells
	CGTCA-motif	CGTCA	646	-	cis-acting regulatory element involved in the MeJA-responsiveness
	CGTCA-motif	CGTCA	1276	-	cis-acting regulatory element involved in the MeJA-responsiveness
	TGACG-motif	TGACG	646	+	cis-acting regulatory element involved in the MeJA-responsiveness
	TGACG-motif	TGACG	1276	+	cis-acting regulatory element involved in the MeJA-responsiveness

Table S2 List of the identified StUBC13 genes and their related information.

Gene Name	Accession number	Protein/AA	Chrom	MW (Da)	pI	Instability index	GRAVY
StUBC35/StUBC13A	Soltu.DM.10G002790	154AA	chr10:2,214,883-2,220,030	17,189.85	6.74	49.90	-0.303
StUBC35/StUBC13A-like	Soltu.DM.01G001370	154AA	chr07:53,662,738-53,668,499	17247.93	6.74	49.90	-0.303

Table S3 Positive clones from yeast two-hybrid screening and information of StUBC13 interaction proteins.

Clone No.	Description	Potato code	AGI code
1	Encodes a protein similar to adenylate kinase (ADK1)	Soltu.DM.03G024600.1	AT5G63400
2	Histone deacetylase 8 (HDA8)	Soltu.DM.06G029470.1	AT1G08460
3	Nascent polypeptide-associated complex (NAC)	Soltu.DM.10G026340.1	AT3G12390
4	UBQ10	Soltu.DM.10G001670.1	AT4G05320
5	RING domain ubiquitin E3 ligase, RGLG1(RING domain ligase 1)	Soltu.DM.09G020590.2	AT5G63970
6	Alkenal/one oxidoreductase (AOR)	Soltu.DM.05G008540.1	AT1G23740
7	Ubiquitin-activating enzyme (E1, UBA1)	Soltu.DM.09G009000.1	AT5G06460
8	RING domain ubiquitin E3 ligase, RGLG2(RING domain ligase 2)	Soltu.DM.03G025320.1	AT5G14420
9	A protein involved in RNA splicing (SUM)	Soltu.DM.07G006360	AT2G26460
10	nuclear factor Y, subunit A6	Soltu.DM.12G026000	AT3G14020
11	Encodes a subunit of CCAAT-binding complex （NFYA1）	Soltu.DM.10G028300.2	AT5G12840
12	Pleckstrin homology (PH) domain-containing protein （SWAP70）	Soltu.DM.05G016700.1	AT2G30880
13	Encodes Ni+ dependent glyoxalase I homolog ATGLX1 (GLX1)	Soltu.DM.02G020230.2	AT1G11840
14	Ribosomal protein S10p/S20e family protein	Soltu.DM.01G035790.1	AT5G62300
15	Encodes a cytosolic phosphoglucomutase (PGM)	Soltu.DM.04G015010.1	AT1G23190
16	chaperone binding / ATPase activator	Soltu.DM.05G016210.1	AT5G58110
17	Encodes a Na+/Ca 2+ exchanger-like protein (NCL)	Soltu.DM.07G023870.1	AT1G53210
18	heat shock protein 70 (HSP70)	Soltu.DM.09G002330.1	AT3G12580
19	EF hand domain protein encodes a calmodulin (CAM7)	Soltu.DM.10G026220.2	AT3G43810
20	NAD(P)-linked oxidoreductase superfamily protein	Soltu.DM.01G050290	AT2G21250
21	Cysteine proteinase precursor-like protein/ dehydration stress-responsive gene (RD21)	Soltu.DM.12G005880.1	AT1G47128
22	Cysteine proteinase precursor-like protein/ dehydration stress-responsive gene (RD21)	Soltu.DM.12G005890.1	AT1G47128
23	Ubiquitin E3 ligase LOG2 (LOSS OF GDU2)	Soltu.DM.09G005970.1	AT3G09770
24	U-box type E3 ubiquitin ligase (PUB40)	Soltu.DM.02G032560.1	AT5G40140
25	MMS ZWEI homologue (UEV1B)	Soltu.DM.04G003350	AT1G70660

Table S4 Sequences of the primers used in this study.

Gene	Forward primer sequence	Reverse primer sequence
Primers for yeast two-hybrid assays		
pGBKT7-StUBC13	CATGGAGGCCGAATTCCCGGGCCC TTAGCGTAAACTCTGCGA	CTAGTTATGCGGCCGCTGCAGTGATA GCAGCTGCAGCAACACT
pGADT7-StLOG2	GCCATGGAGGCCAGTGAATTCATG GGTAATATGGGAAGTAGTGGTG	CAGCTCGAGCTCGATGGATCCTTACT CTTCGGCTGCGCCT
pGADT7-StRGLG1	GCCATGGAGGCCAGTGAATTCATG GGGAATCAAGAGTCTGCC	CAGCTCGAGCTCGATGGATCCCTAGC TATATAGTCTTATGCGAGTTTTAATG
pGADT7-StRGLG2	GCCATGGAGGCCAGTGAATTCATG GGTGGCAAGAGTTCAAAGA	CAGCTCGAGCTCGATGGATCCTCAGT AAAGTCTTATCCTAGTTTGAATACTG
pGADT7-StCAM7	CCATGGAGGCCAGTGAATTCATGG CAGATCAGCTCACCGATG	CGATTCATCTGCAGCTCGAGTCACTT GGCCATCATGAC
pGADT7-StS6PDH	CCATGGAGGCCAGTGAATTCATGG CGATAACACTGAAGAG	CGATTCATCTGCAGCTCGAGTTAGGC ATACAGATCTATG
pGADT7-StUEV1B	CCATGGAGGCCAGTGAATTCATGG GGTCAGAAGGATCATC	CGATTCATCTGCAGCTCGAGTTACAT AATGCAGCATTTC
StUEV1D-like1	GACGTACCAGATTACGCTCATATGA CTCTTGGTTCAGGAGG	CAGCTCGAGCTCGATGGATCCCTAG AAATAGGTGCCTTC
StUEV1D-like	CCATGGAGGCCAGTGAATTCATGA CGCTTGGTTCAGGAGG	CGATTCATCTGCAGCTCGAGGAAAC AGGTGCCTTCGGGTG
StNCL	GACGTACCAGATTACGCTCATATGA TGAAAAAATCTCCAAATATGTTCC	CAGCTCGAGCTCGATGGATCCCTATG ACCAACCAAAGACGTA
StRD21	GACGTACCAGATTACGCTCATATGA TGGCAGCTCACAGCTCAAC	CAGCTCGAGCTCGATGGATCCACTTA CTCTATTGCTTTTGTTGA
StNF-YA6	GACGTACCAGATTACGCTCATATGA TGAAGTCTTTGGATCCGGACT	CAGCTCGAGCTCGATGGATCCTCACC GGACAACTGGAGCAC
Primers for subcellular localization		
StUEV1B-EGFP	GAGCTCGGTACCCGGGGATCCATG GGGTCAGAAGGATCATC	GCCCTTGCTCACCATGTCGACCATAA TGCAGCATTTCACAACTAG
StUBC13-EGFP	GAGCTCGGTACCCGGGGATCCATG GCTAACAGCAATCTTCCTCGA	GCCCTTGCTCACCATGTCGACTCATG CACCACTAGCATATAGGCG

StRGLG1-EGFP	GAGCTCGGTACCCGGGGATCCATG GGGAATCAAGAGTCTGCC	GCCCTTGCTCACCATGTCGACGCTAT ATAGTCTTATGCGAGTTTTAATGG
Primers for BiFC		
pSPYNE-StUEV1B	GGCGCGCCACTAGTGGATCCATGG GGTCAGAAGGATCATC	GCGGTACCCTCGAGGTCGACCATAAT GCAGCATTTC
pSPYCE-StUBC13	GGCGCGCCACTAGTGGATCCATGG CTAACAGCAATCTTCCTCGA	GCGGTACCCTCGAGGTCGACTCATG CACCACTAGCATATAGGCG
pSPYNE-StRGLG1	GGCGCGCCACTAGTGGATCCATGG GGAATCAAGAGTCTGCC	GCGGTACCCTCGAGGTCGACGCTATA TAGTCTTATGCGAGTTTTAATGG
Primers for SLC		
StUEV1B-NLuc	CGAGCTCGGTACCCGGGATCCATG GGGTCAGAAGGATCATC	CGCGTACGAGATCTGGTCGACCATAA TGCAGCATTTCACAACT
StUBC13-CLuc	TACGCGTCCCGGGGCGGTACCATG GCTAACAGCAATCTTCCTCGA	TGGCGCGCCGGGCCCTCTAGATCATG CACCACTAGCATATAGGCG
StRGLG1-NLuc	CGAGCTCGGTACCCGGGATCCATG GGGAATCAAGAGTCTGCC	CGCGTACGAGATCTGGTCGACGCTAT ATAGTCTTATGCGAGTTTTAATGG
